# Synthesis and Characterization of Graft Copolymers with Poly(ε-caprolactone) Side Chain Using Hydroxylated Poly(β-myrcene-*co*-α-methyl styrene)

**DOI:** 10.3390/molecules29102363

**Published:** 2024-05-17

**Authors:** Tao Li, Mingzu Zhang, Jinlin He, Peihong Ni

**Affiliations:** College of Chemistry, Chemical Engineering and Materials Science, State and Local Joint Engineering Laboratory for Novel Functional Polymeric Materials, Jiangsu Key Laboratory of Advanced Functional Polymer Design and Application, Suzhou Key Laboratory of Macromolecular Design and Precision Synthesis, Soochow University, Suzhou 215123, China; 20214209263@stu.suda.edu.cn (T.L.); zhangmingzu@suda.edu.cn (M.Z.); phni@suda.edu.cn (P.N.)

**Keywords:** living anionic polymerization, graft copolymers, β-myrcene, α-methyl styrene, degradable polymer

## Abstract

Graft copolymers have unique application scenarios in the field of high-performance thermoplastic elastomers, resins and rubbers. β-myrcene (My) is a biomass monomer derived from renewable plant resources, and its homopolymer has a low glass transition temperature and high elasticity. In this work, a series of tapered copolymers P(My-*co*-AMS)_k_ (k = 1, 2, 3) were first synthesized in cyclohexane by one-pot anionic polymerization of My and α-methyl styrene (AMS) using sec-BuLi as the initiator. PAMS chain would fracture when heated at high temperature and could endow the copolymer with thermal degradation property. The effect of the incorporation of AMS unit on the thermal stability and glass transition temperature of polymyrcene main chain was studied. Subsequently, the double bonds in the linear copolymers were partially epoxidized and hydroxylated into hydroxyl groups to obtain hydroxylated copolymer, which was finally used to initiate the ring-opening polymerization (ROP) of ε-caprolactone (ε-CL) to synthesize the graft copolymer with PCL as the side chain. All these copolymers before and after modifications were characterized by proton nuclear magnetic resonance (^1^H NMR), gel permeation chromatography (GPC), thermogravimetry analysis (TGA), and differential scanning calorimeter (DSC).

## 1. Introduction

Living and controlled polymerizations play an important role in the field of synthetic polymer chemistry [1,2]. Over the past decades, the discoveries and progresses in this field have allowed researchers to precisely control the chemical structure, molecular weight and molecular weight distribution of polymers. As for styrene (St) and its derivatives, as well as the conjugated 1,3-dienes (isoprene, butadiene, β-myrcene) [3], living anionic polymerization (LAP) has unique advantages in terms of precise control of molecular weight, molecular weight distribution, topology, and microstructure [4,5,6,7]. As an example, styreneic block copolymers are usually synthesized by the sequential addition of monomers from either monofunctional or difunctional initiators [8,9]. In general, they consist of at least three blocks, namely two hard polystyrene end blocks, and one soft mid-block such as polybutadiene or polyisoprene. On a microscopic scale, the soft mid-block acts as a continuous phase providing elasticity, while the two hard end blocks separate domains in the continuous phase, thereby providing physical cross-links. Moreover, block copolymers are usually formed by the connection of two or more homopolymers via covalent or noncovalent bonds. Due to the variance of the chemical structure, repulsion is generally present between different block segments [10]. When the strength of mutual repulsion reaches a certain level, it will induce microphase separation to form nanostructures with various morphologies [11], which endows them with potential applications in a wide range of areas, such as thermoplastic elastomers [12], smart materials [13], drug delivery carriers [14,15], electronics [16], etc.

Thermoplastic elastomers (TPEs) are an important kind of macromolecular material with excellent mechanical properties and low cost, which are used in many aspects of our lives, such as automotive, footwear, adhesives, textiles and biomedical applications [17,18]. In addition to the above-mentioned triblock copolymers, they also have many different topologies such as gradient, star, hyperbranched, dendritic, cyclic and graft [19,20,21,22,23]. Studies on these polymers not only facilitate the exploration of materials with new functionalities and properties, but also improve the ability to tune the properties through chemical design [24]. In particular, graft copolymers with a large number of side chains attached onto a linear backbone are endowed with unusual properties including wormlike conformation, compact molecular dimension and notable chain end effects due to their confined and compact structure compared to linear copolymers with similar molecular weight [25,26,27]. Impressively, the properties of the graft copolymer can be modified by changing the grafting density, length of side chain, and the degree of branching. Polymer chains can be densely tethered through functional groups not only to another polymer chain to form the graft copolymers, but to the surface of a planar, spherical or cylindrical solid which is a distinct area of research in its own right [24]. Regarding how to synthesize graft copolymers, the synthesis methods are based on three main strategies: ‘grafting-onto’, ‘grafting-through’ and ‘grafting-from’ [1]. Among them, the ‘grafting-from’ strategy is to form the side chains from a macromolecular initiator containing many initiating sites. Macromolecular initiators can be synthesized directly from monomers which contain initiating groups, or macromolecules can be synthesized first and then modified with initiating groups. With the advent of polymerization techniques, this strategy is considered a particularly attractive approach for the synthesis of well-defined graft copolymers, limiting the coupling and termination reactions as well as the gradual growth of the side chains encountered in ‘grafting-through’ or ‘grafting-onto’ strategies.

In recent years, with the growing emphasis on environmental protection, polymer chemistry is moving towards sustainability, which includes the development of environmentally friendly production processes and the use of renewable feedstocks instead of fossil fuel sources [28,29,30,31]. Various research teams are already working on preparing novel raw materials from renewable sources and using them to produce new materials [32]. The production of L-lactide from starch and its use in the synthesis of poly(lactic acid) (PLA) is a good example [33] that has well-inspired researchers to extract renewable monomers from starch [34], vegetable oils [35], and cellulose [36]. In this respect, terpenes have attracted much attention due to their ubiquitous nature and high functionality. At present, a variety of terpenes are used to make polyolefins with similar properties as those of natural rubber, such as pinene, limonene, pirocarvone, myrcene, isopropylene, ocimene and farnesene [28]. Among them, β-myrcene and β-farnesene are considered to be promising bio-based monomers that can be used industrially on a large scale to replace isoprene and butadiene. β-myrcene (My) can be obtained by thermal cracking of β-pinene, which is a naturally occurring component of turpentine. β-myrcene has a similar structure to butadiene and isoprene, with high polymerization activity and low polymerization difficulty. Polymyrcene (PMy) with high 1,4-units (>90%), resembling natural rubber, can be prepared by living anionic polymerization (LAP) method in nonpolar solvents [37]. In addition, the microstructures of PMy can be adjusted by tuning the solvent polarity.

However, although PMy is a polymer derived from biomass monomers, it cannot be degraded. α-Methyl styrene (AMS) is a conventional styrenic monomer and its polymer (PAMS) can be broken when heated to 250 °C [38]. Recently, Diao et. al. reported a doping strategy to prepare a recyclable poly(methyl methacrylate) (PMMA) by incorporating a small amount of PAMS units into the polymer backbone, which not only retained the mechanical strength and optical clarity of PMMA, but also endowed the PMMA with thermal depolymerization property [39]. In this work, AMS was used for the first time in the anionic copolymerization with My to obtain P(My-*co*-AMS)_k_ (k = 1, 2, 3) copolymers with different block numbers, in which the PAMS segment would fracture when heated at high temperature and the copolymer thereby can be degraded into low-molecular-weight fractions. Subsequently, the copolymer was epoxidized and hydroxylated into hydroxyl groups, which were then used to initiate the ROP reaction of ε-CL to obtain graft copolymers with PCL as the side chain [40]. The chemical structure, molecular weight and molecular weight distribution of the obtained polymers were characterized by ^1^H NMR and GPC, and their thermal properties were measured by TGA and DSC analyses. This kind of graft copolymer with multiple side hydroxyls is anticipated to exhibit the characteristics of elastomer or can be used as the extender in the preparation of functional polyurethanes.

## 2. Results

### 2.1. Synthesis and Characterization of P(My-co-AMS)_k_ Copolymers

**Scheme 1:**
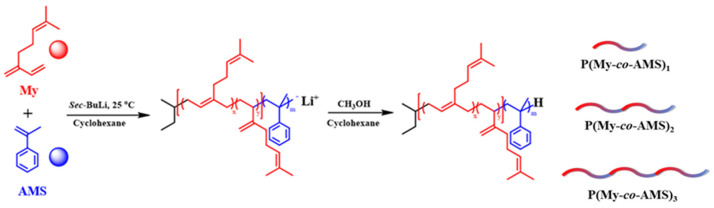
Synthetic route of P(My-co-AMS)_k_ (k = 1, 2, 3) copolymers with different block numbers.

**Figure 1:**
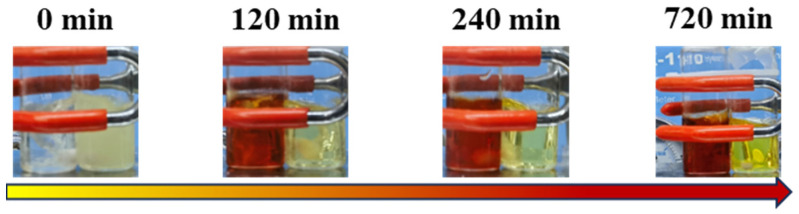
Color change of polymerization solutions at different reaction time: left is copolymerization of My and AMS; right is homopolymerization of My.

**Figure 2:**
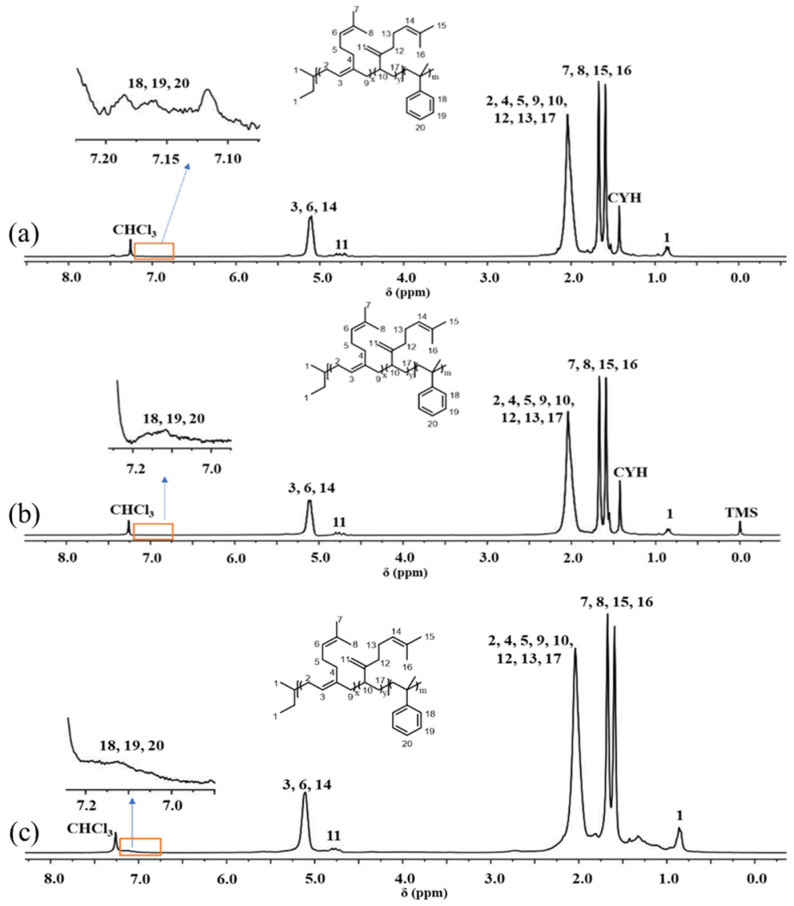
^1^H NMR spectra of P(My-*co*-AMS)_k_ copolymers (solvent: CDCl_3_): (**a**) P(My-*co*-AMS)_1_-3; (**b**) P(My-*co*-AMS)_2_-2; (**c**) P(My-*co*-AMS)_3_-1.

**Figure 3:**
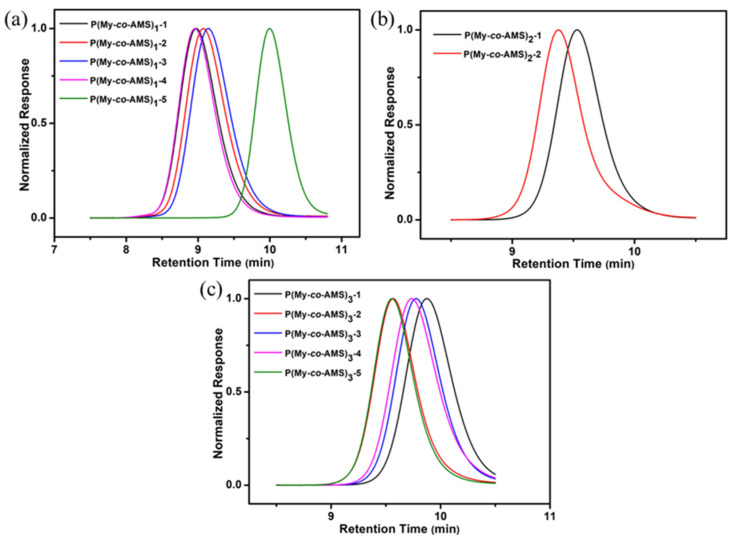
GPC traces of P(My-*co*-AMS)_k_ copolymers: (**a**) P(My-*co*-AMS)_1_; (**b**) P(My-*co*-AMS)_2_; (**c**) P(My-*co*-AMS)_3_.

The synthetic routes of P(My-*co*-AMS)_k_ (k = 1, 2, 3) copolymers are shown in Scheme 1. From previous reports [37], it is known that the PMy chain obtained in cyclohexane is dominated by the 1,4-units (94%) and a small amount of 3,4-units (6%). A series of P(My-*co*-AMS)_k_ copolymers were prepared by LAP in cyclohexane using sec-BuLi as initiator at room temperature (see details in Section 3). The color change of solutions for homopolymerization of My, copolymerization of My and AMS with reaction time are shown in Figure 1. It can be obviously found that the color of the copolymerization solution (left bottle) gradually changed from yellow color of the PMy^-^Li^+^ living chain to red color of the PAMS^-^Li^+^ living chain, while the homopolymerization solution (right bottle) kept yellow from the beginning to the end. Figure 2 shows the ^1^H NMR spectra of representative P(My-*co*-AMS)_k_, the characteristic proton peaks can be attributed as follows: δ = 0.66–0.87 (1, C***H***_3_CH_2_-, C***H***_3_CH-), δ = 2.02 (2, 4, 10 -CHC***H***_2_C-, -CC***H***_2_C***H***_2_-, -CH_2_C***H***CH_2_), δ = 1.66, 1.58 (7, 8, -CC***H***_3_), δ = 4.66–4.80(11, -CC***H***_2_), δ = 5.10 (3, 6, 14, -CC***H***-), δ = 7.00–7.20 (18, 19, 20, -CHC_6_***H***_5_-). The molecular weights and molecular weight distributions of copolymers were characterized by GPC, and the results are shown in Figure 3 and Table 1. All the copolymers showed a single peak distribution with narrow molecular weight distribution (*Ð* = 1.09–1.15). Combining the results of GPC and ^1^H NMR tests, it can be concluded that the P(My-*co*-AMS)_k_ copolymers with different molecular weights have been successfully synthesized.

**Figure 4:**
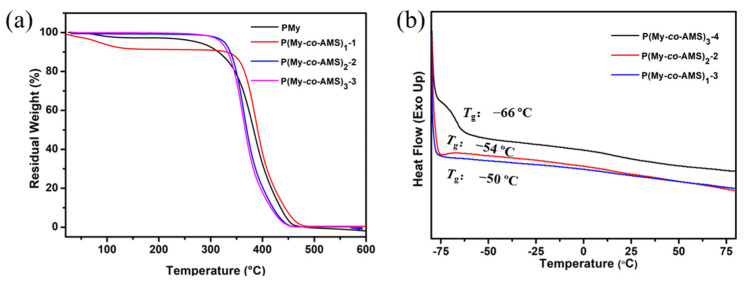
(**a**) TGA thermograms and (**b**) DSC thermograms of P(My-co-AMS)_k_.

After a small amount of PAMS was added to PMy, and the glass transition temperature (*T*_g_) of PAMS is as high as 168 °C, in order to investigate the thermal stability and *T*_g_ of the main chain after the addition of PAMS unit, the PMy homopolymer and its copolymer with PAMS were tested by TGA and DSC. As shown in Figure 4a, it can be observed from the TGA curve of PMy that there is a loss of weight at 100 °C, which is due to the residual moisture in the sample. The rapid decomposition temperature (*T*_d_), which is the temperature corresponding to the lowest point after the first order derivation of the heat loss curve, was recorded from the curves and is shown in Table 2. It is found that the thermal decomposition temperatures of the linear copolymers and the homopolymer were very close to each other. This may be due to the small amount of AMS incorporation and the short PAMS chain segments resulting in the absence of an obvious thermal weight loss plateau, which in turn leads to an insignificant change in the thermal decomposition temperature. In addition, it can be seen from the figure that the thermal decomposition temperature of the linear copolymers was basically unchanged even if the number of copolymerization segments changes, which indicates that the thermal stability of the linear multiblock copolymer is not much related to the number of copolymerization segments with a small molecular weight difference, and that the addition of a small amount of AMS did not affect the thermal stability of the polymer chain significantly. It is known from the literature that the *T*_g_ value of PMy homopolymer is around −64 °C [41]. The DSC curves of linear copolymers are shown in Figure 4b, from which it can be seen that the *T*_g_ values of copolymers remains in this interval, indicating that the doping of a small amount of AMS did not significantly affect the flexibility of the PMy backbone. However, due to the low content of PAMS chain segments and high content of PMy chain in the sample, only the *T*_g_ of PMy can be found in the curve, while it is difficult to observe the *T*_g_ of PAMS.

### 2.2. Thermal Degradation of P(My-co-AMS)_k_ and PMy

**Figure 5:**
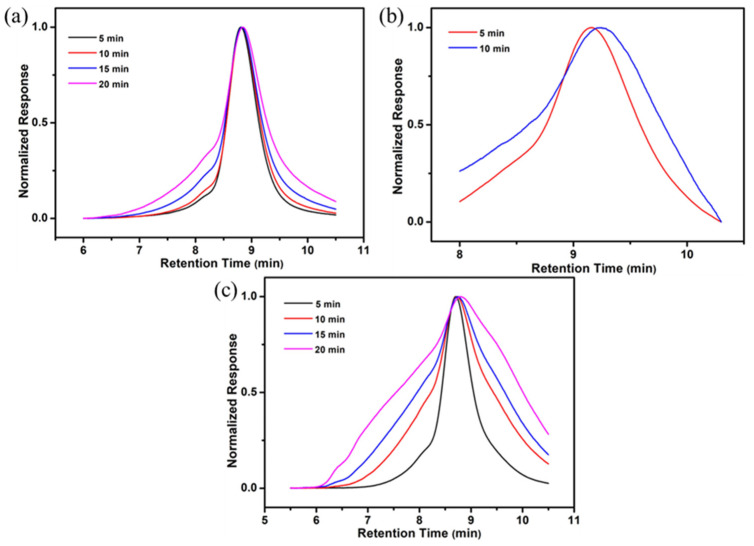
GPC traces of polymer samples after heating for different times. (**a**): PMy; (**b**): P(My-*co*-AMS)_1_-3; (**c**): P(My-*co*-AMS)_2_-2.

In this study, a simple heating degradation experiment under reduced pressure was carried out on PMy homopolymer and linear copolymers, where the polymers were placed under vacuum and heated (250 °C) for different times and then sampled at different times for GPC test to observe the molecular weight changes. The GPC curves of the samples after heating the PMy homopolymer for different times are shown in Figure 5a, from which it can be seen that PMy has a better stability after heating, and the main peak is basically not changed. However, with the increasing heating time, the proportion of the insoluble part of the sample increased, while the GPC curve measured for the soluble part became wider. After doping a small number of PAMS units in the PMy backbone, the linear copolymer P(My-*co*-AMS)_1_ was heated, and the GPC curves of the products after heating for different times are shown in Figure 5b. It can be observed from the figure that unlike PMy homopolymer, the PAMS fragments in P(My-*co*-AMS)_1_ have undergone thermal degradation, which resulted in very broad peaks on the GPC curves after heating. Moreover, the linear copolymer P(My-*co*-AMS)_2_ with two block numbers was also heated and the GPC curves of samples after heating for different times are shown in Figure 5c. It can be also observed that a very broad peak occurred on the GPC curves after heating due to the presence of PAMS.

### 2.3. Synthesis and Characterization of E[P(My-co-AMS)_k_]

**Scheme 2:**
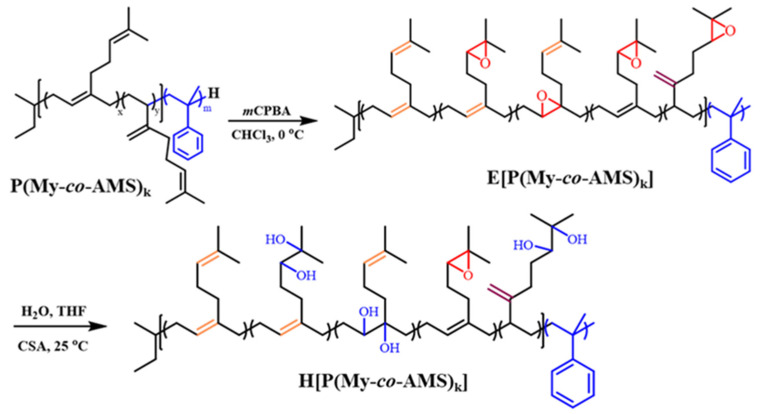
Synthetic routes of E[P(My-*co*-AMS)_k_] and H[P(My-co-AMS)_k_].

**Figure 6:**
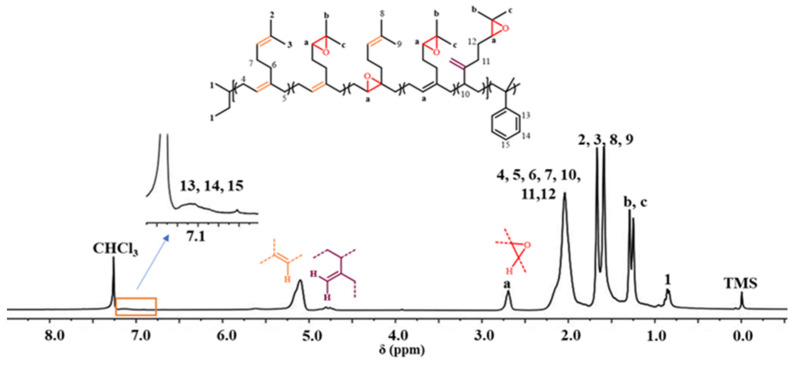
^1^H NMR spectrum of E[P(My-*co*-AMS)_3_]-3 (solvent: CDCl_3_).

The synthetic routes for epoxidation and hydroxylation reactions are shown in Scheme 2. First, the polymer was reacted with meta-chloroperoxybenzoic acid (mCPBA) ([mCPBA]/[C=C] = 0.30) in chloroform at 0 °C for 1 h to obtain a quantitatively epoxidized P(My-co-AMS)_3_. The ^1^H NMR spectrum of the product is shown in Figure 6. The attribution of some of the proton peaks can be found in the spectrum as follows: δ = 0.66–0.87 (1, C**H**_3_CH_2_-, C**H**_3_CH-), δ = 2.02 (-CHC**H**_2_C-, -CC**H**_2_C**H**_2_-, -CH_2_C**H**CH_2_), δ = 1.66, 1.58 (2, 3, 8, 9, -C(C**H**_3_)_2_), δ = 4.66–4.80 (-CC**H**_2_), δ = 5.10 (-CC**H**-), δ = 7.00–7.20 (13, 14, 15, -CHC_6_**H**_5_-). In addition to the same characteristic peaks as previously described for P(My-co-AMS)_3_, the characteristic peaks of two methyl (b, c) protons in the epoxide group were observed at δ = 1.25 and 1.29, respectively, and the characteristic peak of a hypromethyl proton in the epoxide group (a) was found at δ = 2.69 [41]. The presence of these two characteristic peaks can prove that part of the double bond in the polymer chain was successfully converted into epoxy group, indicating the successful epoxidation reaction. The epoxidation degree of about 20–30% can be calculated from the integral area ratios of the three peaks at δ = 2.69, 4.66–4.80 and 5.10.

**Figure 7:**
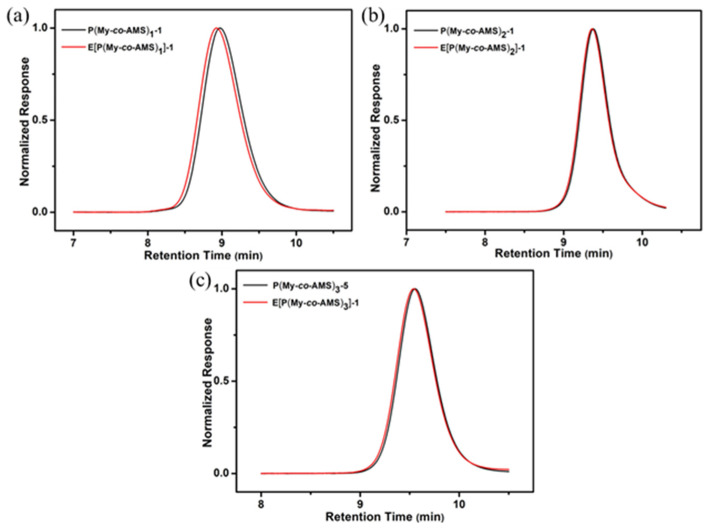
GPC traces of E[P(My-*co*-AMS)_k_] and its precursors: (**a**) E[P(My-*co*-AMS)_1_]-1; (**b**) E[P(My-*co*-AMS)_2_]-1; (**c**) E[P(My-*co*-AMS)_3_]-1.

GPC was used to characterize the molecular weights and molecular weight distributions of E[P(My-*co*-AMS)_k_], the GPC curves are shown in Figure 7 and the relevant data are summarized in Table 3. It can be seen from Figure 7 that all the GPC curves are single-peak distributions with low molecular weight distributions (*Ð* = 1.05–1.15). Moreover, the *Ð* values of the epoxidation products were not broadened compared to their corresponding precursor polymers, and no shoulder peaks were observed. Finally, from the molecular weight point of view, it was found that the molecular weight of the epoxidation product increased slightly, which was consistent with the epoxidation results. Combined with the result of ^1^H NMR spectrum, it can be proved that the epoxidation reaction was successfully carried out and the epoxidized copolymer can be used in the next step of the reaction.

### 2.4. Synthesis and Characterization of HP(My-co-AMS)_3_

In general, epoxides can react with nucleophilic reagents under acidic or basic conditions. Among the most prominent reactions, epoxides are treated with diamines to obtain cured epoxy resins or with thiolates to produce functionalized polymers [41]. Schlaad found that 49% epoxidized PMy is stable under basic conditions without reacting with nucleophilic reagents in solution [41]. On the other hand, the polymers readily crosslinked in DCM solution after treatment with strong acids (H_2_SO_4_) or Lewis acids at room temperature. It is hypothesized that this is due to the presence of trace amounts of water in the system. The authors concluded that hydrolysis should be preferred and cross-linking avoided when increasing the concentration of water. Therefore, an aqueous solution of CSA is used to hydrolyze 49% epoxidized 1,4-PMy in THF solution (water/THF = 1:4 *v*/*v*) at room temperature.

**Figure 8:**
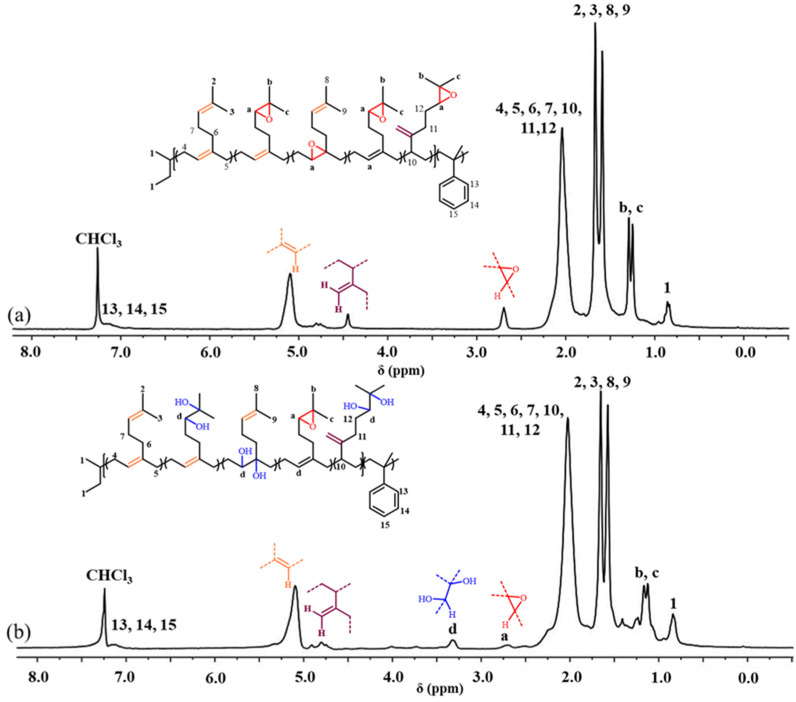
^1^H NMR spectra of H[P(My-*co*-AMS)_3_]-2 and its precursors: (**a**) E[P(My-*co*-AMS)_3_]-4; (**b**) H[P(My-*co*-AMS)_3_]-2. (solvent: CDCl_3_).

In the present study, as a demonstration, the hydroxylation reaction of E[P(My-*co*-AMS)_3_] was also carried out by the same method [41]. The ^1^H NMR spectra of H[P(My-*co*-AMS)_3_] and its precursor are shown in Figure 8, from which it can be clearly seen that the characteristic peak attributed to the epoxy group proton at δ = 2.69 was significantly weakened. A characteristic peak belonging to the proton on the carbon attached to the hydroxyl group appeared at δ = 3.3 ppm. The rest of the characteristic peaks are consistent with those described in the previous section, demonstrating the successful synthesis of hydroxylated product.

### 2.5. Synthesis and Characterization of P(My-co-AMS)_3_-g-PCL

**Scheme 3:**
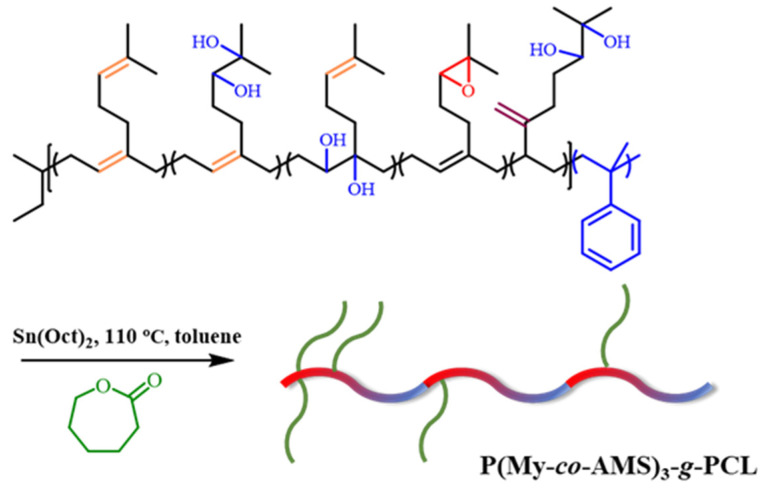
Synthetic route of P(My-*co*-AMS)_3_-*g*-PCL.

The synthetic route of P(My-*co*-AMS)_3_-*g*-PCL graft copolymer is shown in Scheme 3. Li et al. performed the ring-opening polymerization (ROP) of ε-CL at room temperature using side hydroxyl groups on the main chain of hydroxylated polybutadiene as initiator and TBD as catalyst [42]. However, in this experiment, TBD failed to initiate the ROP of ε-CL at room temperature. Instead, we used stannous octanoate as the catalyst and conducted the ROP reaction at 90 °C, 100 °C, and 110 °C, respectively. The reaction was successfully performed at 110 °C.

**Figure 9:**
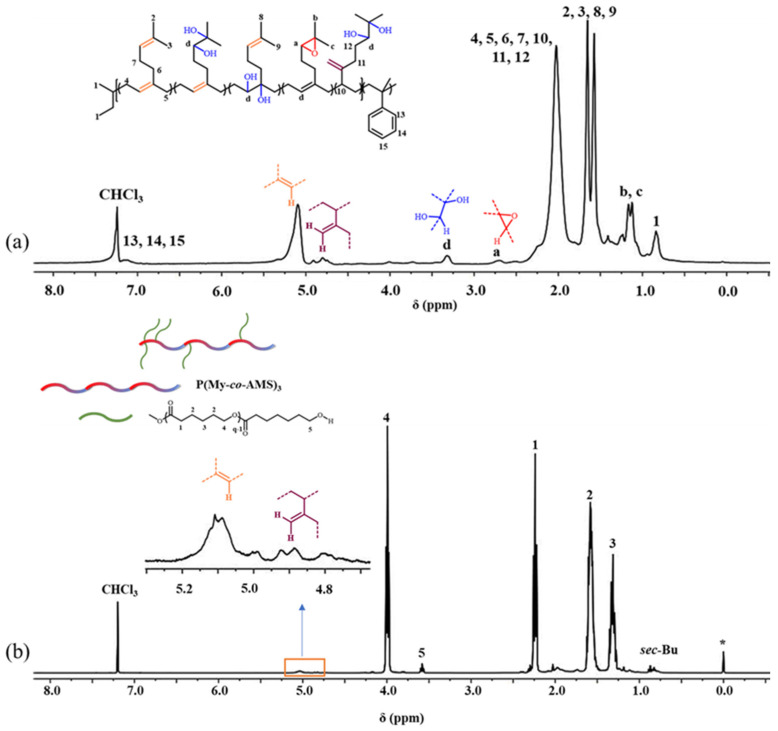
^1^H NMR spectra of (**a**) H[P(My-*co*-AMS)_3_]-2 and (**b**) P(My-*co*-AMS)_3_-*g*-PCL-2. (solvent: CDCl_3_, *: silicone grease).

The molecular weights of the macromolecular initiators used were all similar, only changing the proportions of monomers and hydroxyl groups. All samples were synthesized under the same reaction conditions except for the macroinitiators, which have similar molecular weights and are epoxidized to a similar extent, so that the differences in the molecular weights of the polymers mainly originate from differences in the molecular weights of the single arm. Subsequently, ^1^H NMR and GPC tests were performed to verify the successful synthesis of the graft copolymers. A series of graft copolymers were prepared by initiating the ROP of ε-CL using a linear hydroxylated copolymer as initiator and Sn(Oct)_2_ as catalyst. Taking the graft copolymer P(My-*co*-AMS)_3_-*g*-PCL-2 as an example, its chemical structure was characterized using ^1^H NMR, and the results are shown in Figure 9. It can be clearly seen that after the ROP reaction, the characteristic proton peak attributed to the hydroxyl group on the adjacent carbon atom at δ = 3.3 disappears, while the methylene proton peak attached to the hydroxyl group at the end of the PCL chain appears at δ = 3.65 (signal 5 in Figure 9b). In addition, the characteristic methylene proton peaks on the PCL chain can be found at δ = 4.05, 2.25, 1.64, and 1.37. In addition, the peaks of PMy backbone between δ = 1–2 can also be observed along with a small number of characteristic peaks of residual double bond protons of PMy backbone at δ = 4.8–5.2.

**Figure 10:**
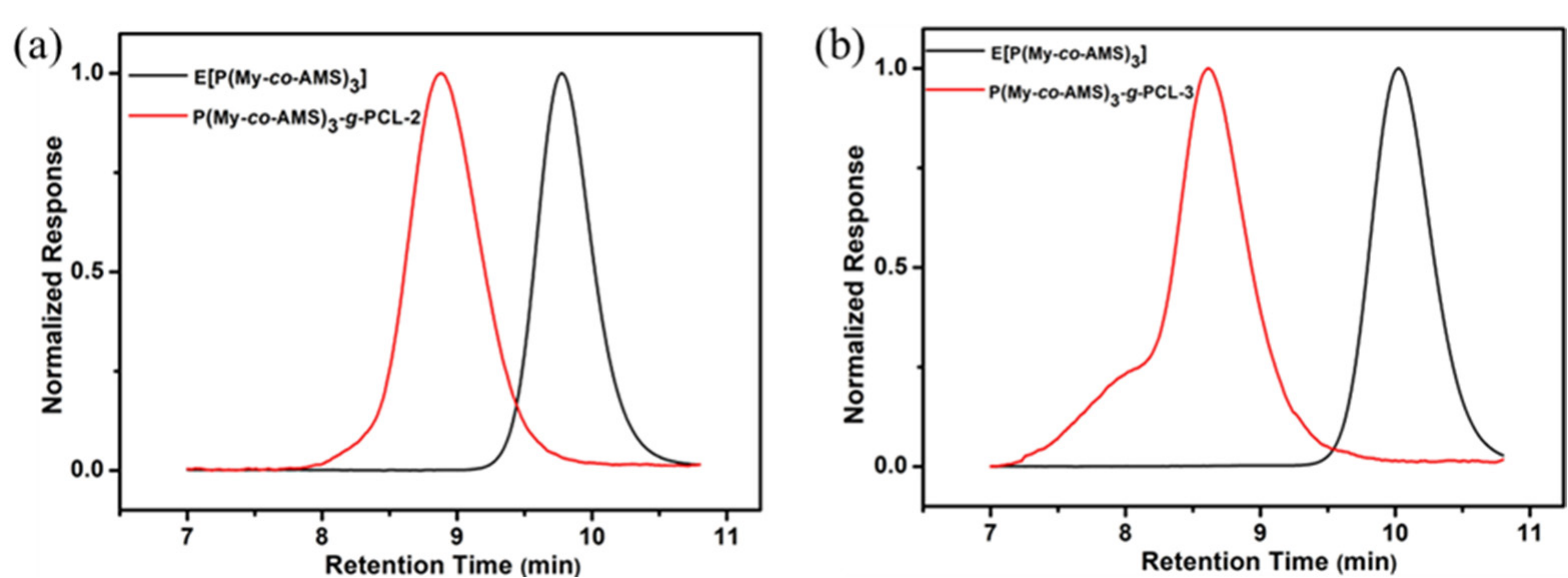
GPC traces of graft copolymers and their precursors: (**a**) P(My-*co*-AMS)_3_-*g*-PCL-2; (**b**) P(My-*co*-AMS)_3_-*g*-PCL-3.

The graft copolymers were further characterized by GPC analysis, and the results are shown in Figure 10 and Table 4. From the figure, it can be seen that the elution curves of the graft copolymers were obviously shifted to the direction of high molecular weight compared with those of the precursor copolymers. Meanwhile, the GPC curves of the graft copolymers basically showed a single-peak distribution with a relatively narrow molecular weight distribution (*Ð* = 1.10–1.25), except for a small number of shoulder peaks at high molecular weight in some samples. In addition, since both the epoxidized copolymer and the hydroxylated copolymer were insoluble in methanol, if the hydroxylated copolymer was not involved in the initiation of the ROP of ε-CL, an obvious peak belonging to the precursor copolymer would appear in the direction of the low molecular weight in the GPC curves of the graft copolymers, which further proved that the hydroxylated copolymers had successfully initiated the ROP of ε-CL.

**Figure 11:**
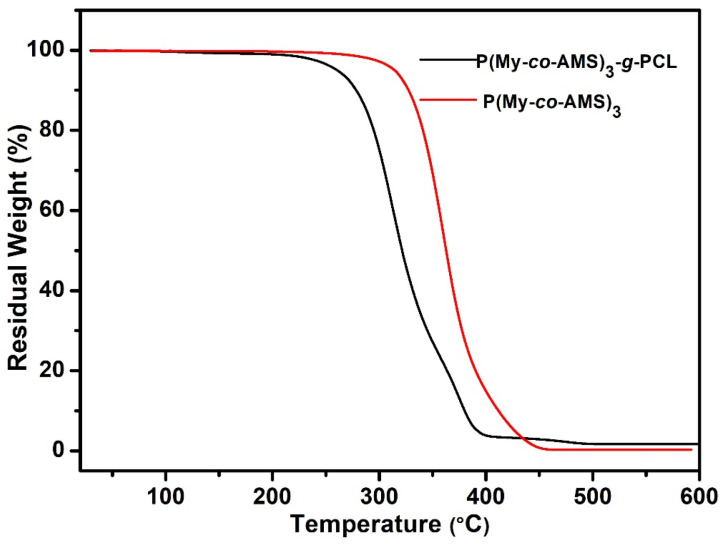
TG curves of graft copolymer and its precursor.

**Figure 12:**
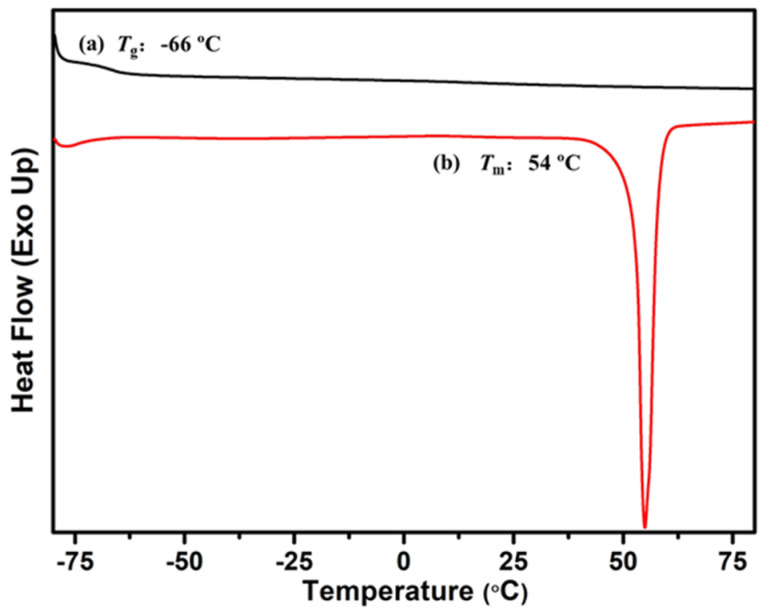
DSC thermograms of (**a**) P(My-*co*-AMS)_3_ and (**b**) P[(My-*co*-AMS)_3_]-*g*-PCL.

Figure 11 shows the TGA curves of graft copolymer P(My-*co*-AMS)_3_-g-PCL. It can be observed from the figure that the TGA curve of graft copolymers shows only one stage of weight loss, and its rapid decomposition temperature (*T*_d_) is about 305 °C, which was significantly decreased compared to the corresponding precursor copolymer (360 °C). Subsequently, the graft copolymer and its precursor were characterized using DSC and the result is shown in Figure 12. As can be seen from the figure, the P(My-*co*-AMS)_3_ has a distinct *T*_g_ attributed to the PMy backbone at around −66 °C, whereas the graft copolymer P(My-*co*-AMS)_3_-g-PCL shows only one melting peak attributed to the PCL chain segment at around 54 °C.

## 3. Materials and Methods

### 3.1. Starting Materials

*sec*-Butyllithium (sec-BuLi, 1.3 mol L^−1^ in *n*-hexane, Shangyu Hualun Chemical Industry, Shaoxing, China) (CAUTION: highly flammable), calcium hydride (CaH_2_, A.R., Aladdin, Shanghai, China), 3-chloroperbenzoic acid (*m*CPBA, 75%, Aladdin, Shanghai, China), tetrahydrofuran (THF, A.R., Enox, Changshu, China), and camphorsulfonic acid (CSA, 98%, TCI, Tokyo, Japan) were used directly. As the termination reagent, methanol (CH_3_OH, A.R., Enox, Changshu, China) was firstly purified by stirring in CaH_2_ powder for 12 h at room temperature and degassed on a vacuum line. It was subsequently distilled into a Schlenk flask and stored in a glove box. The methanol applied in other experimental procedures was used directly. β-myrcene (My, 90%, Xiya Reagent, Linyi, China), α-methyl styrene (AMS, 99%, Energy Chemical, Shanghai, China) and cyclohexane (CYH, A.R., Enox, Changshu, China) were purified using the method described above for purifying methanol. Toluene (A.R., Enox, Changshu, China), chloroform (CHCl_3_, A.R., Enox, Changshu, China), stannous octoate (Sn(Oct)_2_, 95%, Adamas-Beta, Shanghai, China) and ε-caprolactone (ε-CL, 97%, Aladdin, Shanghai, China) was distilled under reduced pressure before use.

### 3.2. Synthesis of P(My-co-AMS)_k_ Copolymers

Anionic polymerization is extremely sensitive to moisture and oxygen. The monomers and solvents involved in this experiment need to be treated with high vacuum lines. All the polymerization reactions in this paper were carried out in a glovebox (<0.2 ppm O_2_, <0.1 ppm H_2_O) filled with argon. The synthetic routes of P(My-*co*-AMS)_k_ (k = 1, 2, 3) copolymers with different block numbers are shown in Scheme 1. Taking P(My-co-AMS)_3_-4 as an example, the measured amount of sec-BuLi (0.7 mL, 0.91 mmol) and pretreated cyclohexane (5 mL) were added into the reaction flask at 25 °C. The mixed monomers of AMS (0.5 mL, 0.45 g, 3.84 mmol) and My (3 mL, 2.37 g, 17.39 mmol) were then added to the reaction flask under stirring in three equal portions, each at an interval of 12 h to obtain the living copolymer chain P(My-*co*-AMS)_3_^-^Li^+^. At the end of the reaction, the reaction was terminated by the addition of degassed methanol. The solution was concentrated and precipitated in ice methanol to obtain the copolymer (1.54 g, yield: 54%). For the synthesis of P(My-*co*-AMS)_1_ and P(My-*co*-AMS)_2_ copolymers, the mixed monomers of AMS and My were added to the reaction flask under stirring in one pot or two equal portions.

### 3.3. Thermal Degradation of P(My-co-AMS)_k_ Copolymers

An appropriate amount of PMy homopolymer or P(My-*co*-AMS)_k_ copolymers was heated at 250 °C under reduced pressure. The samples taken out at different time intervals were subjected to GPC test.

### 3.4. Epoxidation of P(My-co-AMS)_k_

The epoxidized copolymers with different degrees of epoxidation can be obtained by controlling the ratio of mCPBA and C=C [41]. Take E[P(My-*co*-AMS)_3_]-1 for example. P(My-*co*-AMS)_3_ (0.5 g, 0.13 mmol, 7.3 mmol C=C) was dissolved in dry CHCl_3_ (15 mL) in a flask, then the flask was transferred to a 0 °C bath. After cooling down, a quantitative amount of *m*CPBA (0.5 g, 2.1 mmol) ([*m*CPBA]0/[C=C]0 = 0.30) was added to the reaction flask in three portions, and finally the reaction was continued at 0 °C for 1 h. After that, the reaction flask was removed from the bath and after returning to room temperature, it was quenched by the addition of saturated aqueous Na_2_SO_3_ solution under vigorous stirring. The organic layer was separated and washed three times with 1 mol L-1 aqueous NaOH solution. The solvent was evaporated under reduced pressure and the polymer was dried under vacuum until constant weight (0.4 g, yield: 80%).

### 3.5. Hydrolysis of EP(My-co-AMS)_3_

E[P(My-*co*-AMS)_3_] (0.10 g, 0.44 mmol) was weighed and dissolved in 2 mL of THF and added to a flask, while camphor sulfonic acid (CSA) (15 mg, 0.03 mmol) was dissolved in 0.5 mL of deionized water. An aqueous solution of CSA was added with stirring and stirred at room temperature for 24 h. At the end of the reaction, an appropriate amount of CHCl3 was added and the organic layer was washed to neutrality with deionized water. The organic solvent was evaporated under reduced pressure and the polymer was dried under vacuum until constant weight to obtain the hydroxylated product H[P(My-*co*-AMS)_3_]-1 (0.05 g, yield: 50%).

Before ring-opening polymerization, the hydroxylated product was added to the flask and dissolved with dry THF. The residual water in the macromolecular initiator was removed by azeotropic distillation, which was repeated 2–3 times and the refined product was stored in a desiccator at room temperature.

### 3.6. Ring-Opening Polymerization of ε-CL with H[P(My-co-AMS)_3_]

All experimental devices were dried at high temperatures to remove water. As shown in Scheme 3, the ring-opening polymerization of ε-CL was initiated by the side hydroxyl groups on H[P (My-*co*-AMS)_3_] and catalyzed by Sn(Oct)_2_ at 110 °C. Under the protection of nitrogen, anhydrous toluene (10 mL, 93.4 mmol), Sn(Oct)_2_ (0.2 g, 0.49 mmol), macromolecular initiator (50 mg, 0.22 mmol) and ε-CL (1.08 g, 9.5 mmol) were sequentially added to the reaction flask. After the feeding was completed, the reaction bottle was frozen with liquid nitrogen, pumped for 10 min after complete freezing, thawed and filled with nitrogen, and the reaction was then carried out at 110 °C for 8 h. At the end of the reaction, the reaction flask was removed from the oil bath and allowed to return to room temperature before adding 0.5 mL of glacial acetic acid. Finally, the solution was concentrated, precipitated in ice methanol, filtered, and dried under vacuum until constant weight to obtain the graft copolymer P(My-*co*-AMS)_3_-*g*-PCL. (0.57 g, yield: 52%).

### 3.7. Characterization

#### 3.7.1. Nuclear Magnetic Resonance (NMR) Analysis

^1^H NMR spectra were obtained on a 400 MHz spectrometer (Bruker Avance Neo, 400 MHz, Karlsruhe, Germany) using approximately 11 mg of the sample dissolved in 0.6 mL of CDCl_3_.

#### 3.7.2. Gel Permeation Chromatography (GPC)

GPC for analyzing molecular weights and molecular weight distributions of polymers was performed using an HLC-8320 (TOSOH, Yamaguchi, Japan) with THF as the eluent at a flow rate of 1.0 mL min^−1^ at 40 °C. The sample (6 mg) was dissolved in 3 mL of THF, filtered through a 0.22 μm filter and added to a 2 mL sample vial. A series of polystyrene with different molecular weights were used as the standards.

#### 3.7.3. Thermogravimetry Analysis (TGA)

TGA was performed on an instrument (Discovery, TA, New Castle, DE, USA) with about 5 mg of the sample in the platinum plate. Beginning with equilibrating at 30 °C, the temperature was ramped from 30 to 600 °C at a heating rate of 10 °C min^−1^ under a nitrogen atmosphere.

#### 3.7.4. Differential Scanning Calorimeter (DSC)

DSC curves were obtained using a DSC 2010 instrument (TA, New Castle, DE, USA) from −80 to 200 °C at a heating rate of 10 °C min^−1^ with a 5 min isothermal hold at the maximum and minimum temperatures. All glass transition temperatures (*T*_g_) of the samples were reported according to the second heating scans.

## 4. Conclusions

We used anionic polymerization to produce a linear copolymer P(My-*co*-AMS)_k_ based on AMS and My. Successful preparation of the copolymer with a narrow molecular weight distributions (*Ð* < 1.10) was demonstrated by ^1^H NMR and GPC characterizations. The thermal properties of the copolymers were characterized by DSC and TGA. The heating degradation experiment was conducted on the copolymer, and it was found that the GPC curves of the copolymers would shift, and the molecular weight distribution was broadened, which initially showed that PAMS in the polymer chain played a certain role in thermal decomposition. In addition, the macromolecular initiator was obtained by partly transforming the double bond in the main chain of the linear copolymer into the epoxy group, which was then converted into hydroxyl groups by acid-catalyzed hydrolysis. The successful synthesis of epoxidized and hydroxylated copolymers was verified by the appearance of characteristic peaks in the ^1^H NMR spectra. The hydroxylated copolymer was used to initiate the ROP of ε-CL to prepare the graft copolymer P(My-*co*-AMS)_3_-g-PCL with PCL in the side chain. The successful synthesis of graft copolymer was demonstrated by ^1^H NMR analysis, and the GPC curves basically showed a single-peak distribution with relatively narrow molecular weight distribution (*Ð* < 1.25). The results of TGA tests showed that the incorporation of PCL in the side chain made the graft copolymer have a lower thermal decomposition temperature than the precursor copolymer. In addition, the melting peak belonging to PCL appeared in the DSC curve at about 54 °C.

## Figures and Tables

**Table 1:** Molecular weight information of P(My-*co*-AMS)_k_ copolymers.

Sample	Entry	$\bar{M}$_n, GPC_ (kg mol^−1^) *^a^*	$\bar{M}$_w, GPC_ (kg mol^−1^) *^a^*	*Ð ^a^*
P(My-*co*-AMS)_1_-1	20231127-1	3.70	4.10	1.09
P(My-*co*-AMS)_1_-2	20231127-2	4.10	4.50	1.08
P(My-*co*-AMS)_1_-3	20231123-1	4.20	4.60	1.08
P(My-*co*-AMS)_1_-4	20231123-2	3.40	3.80	1.09
P(My-*co*-AMS)_1_-5	20230425	1.80	2.00	1.12
P(My-*co*-AMS)_2_-1	20230516-2	3.90	4.40	1.12
P(My-*co*-AMS)_2_-2	20230504-2	4.80	5.60	1.15
P(My-*co*-AMS)_3_-1	20230523-1	2.20	2.50	1.13
P(My-*co*-AMS)_3_-2	20230523-2	3.70	4.10	1.13
P(My-*co*-AMS)_3_-3	20230516-1	2.50	2.90	1.13
P(My-*co*-AMS)_3_-4	20230530-2	2.70	3.10	1.14
P(My-*co*-AMS)_3_-5	20230509-2	3.80	4.20	1.12

*^a^* Determined by GPC with THF as the eluent and PS as standards.

**Table 2:** TGA data of PMy and P(My-*co*-AMS)_k_.

Sample	Entry	*T*_d_ (°C)
PMy	20230504-1	375
P(My-*co*-AMS)_1_-1	20231127-1	381
P(My-*co*-AMS)_2_-2	20230504-2	370
P(My-*co*-AMS)_3_-3	20230516-1	362

**Table 3:** Summary of molecular weight data for E[P(My-*co*-AMS)_k_].

Sample	Entry	$\bar{M}$_n, GPC_ (kg mol^−1^) *^a^*	$\bar{M}$_w, GPC_ (kg mol^−1^) *^a^*	*Ð ^a^*
E[P(My-*co*-AMS)_1_]-1	20231201	4.30	4.80	1.08
E[P(My-*co*-AMS)_2_]-1	20230515	4.80	5.70	1.12
E[P(My-*co*-AMS)_3_]-1	20230517	3.9	4.4	1.11
E[P(My-*co*-AMS)_3_]-2	20230530	3.7	4.2	1.13
E[P(My-*co*-AMS)_3_]-3	20230531	2.2	2.5	1.12
E[P(My-*co*-AMS)_3_]-4	20230609	2.7	3.1	1.12

*^a^* Determined by GPC with THF as the eluent and polystyrene as standards.

**Table 4:** Summary of molecular weight data for P(My-*co*-AMS)_3_-*g*-PCL and its precursor.

Sample	Entry	$\bar{M}$_n, GPC_ (kg mol^−1^) *^a^*	$\bar{M}$_w, GPC_ (kg mol^−1^) *^a^*	*Ð ^a^*
E[P(My-*co*-AMS)_3_]	20230822	2.50	2.90	1.13
P(My-*co*-AMS)_3_-*g*-PCL-2	20231230-1	4.70	5.20	1.10
E[P(My-*co*-AMS)_3_]	20230609	2.70	3.10	1.15
P(My-*co*-AMS)_3_-*g*-PCL-3	20231227	6.70	8.10	1.25

*^a^* Determined by GPC with THF as the eluent and polystyrene as standards.

## Data Availability

Data are contained within the article.
